# A novel rabbit monoclonal antibody platform to dissect the diverse repertoire of antibody epitopes for HIV-1 Env immunogen design

**DOI:** 10.1186/1742-4690-9-S2-P83

**Published:** 2012-09-13

**Authors:** Y Chen, M Vaine, X Kong, D Montefiori, S Wang, S Lu

**Affiliations:** 1 University of Massachusetts Medical School, Worcester, MA, USA; 2 New York University School of Medicine, New York, NY, USA; 3 Duke University Medical Center, Durham, NC, USA

## Background

The majority of available monoclonal antibodies (mAbs) in the current HIV vaccine field are generated from HIV-1 infected people. In contrast, preclinical immunogenicity studies have mainly focused on polyclonal antibody responses in experimental animals. Although rabbits have been widely used for antibody studies, there has been no report of using rabbit mAbs to dissect the specificity of antibody responses for AIDS vaccine development.

## Methods

Here we report the production of a panel of 12 mAbs from one NZW rabbit that was immunized with a HIV-1 JR-FL gp120 DNA prime and protein boost vaccination regimen.

## Results

These rabbit mAbs recognized a diverse repertoire of epitopes. Besides the traditional highly immunogenic V3 region, these mAbs recognized several previously underappreciated epitopes in the C1, C4, and C5 regions. Nine mAbs showed cross-reactivity against gp120s of clades other than clade B. At least three mAbs showed neutralizing activities with various breadth and potency. Increased somatic mutation percentage and long CDR3 were observed with some of the rabbit mAbs. More interestingly, phylogenic tree analysis showed that the heavy chain of mAbs recognizing the same region on gp120 were segregated into an independent subtree, implicating that these mAbs may derive from the same B cell precursor. Crystal structures of several rabbit mAbs suggested that these rabbit mAbs generated from vaccines mimic the binding modes of well-characterized human mAbs isolated from infected individuals.

## Conclusion

Therefore, isolation of mAbs from vaccinated rabbits provides us an opportunity to study the evolution and affinity maturation of HIV-1 Env-specific mAbs elicited by candidate AIDS vaccines.

